# Roles of endovascular aneurysm repair in management of secondary aorto-enteric fistulas

**DOI:** 10.1093/jscr/rjaa520

**Published:** 2020-12-26

**Authors:** Barnaby Jmc Farquharson, Leanna Erete, Luke Morgan-Rowe, Matthew Metcalfe

**Affiliations:** Lister Hospital, Stevenage, East and North Hertfordshire NHS Trust, London, UK; Lister Hospital, Stevenage, East and North Hertfordshire NHS Trust, London, UK; Lister Hospital, Stevenage, East and North Hertfordshire NHS Trust, London, UK; Lister Hospital, Stevenage, East and North Hertfordshire NHS Trust, London, UK

## Abstract

Secondary aorto-enteric fistulas (AEFs) are an uncommon but serious complication of abdominal aortic aneurysm (AAA) repair. Case review of two cases of secondary AEF are as follows: the first case involved a 75-year- old male who presented with AEF 1 year post-emergency open AAA repair, successfully managed with endovascular aortic aneurysm repair (EVAR) without complication. The second case involved a 75-year-old male patient 14 months post open AAA repair for an inflammatory aneurysm who presented with an iliac-enteric fistula. The previous repair was relined with a bifurcated EVAR with subsequent laparotomy and resection of the affected portion of the small bowel. Both patients on lifelong antibiotics without further episodes of sepsis, recurrence of AEF or mortality at 12 months follow up. EVAR can be successful in the management of secondary AEF. Careful patient selection, accurate image interpretation, and expedient management are key factors to successful short- and long-term outcomes.

## BACKGROUND

Aorto-enteric fistula (AEF) is an abnormal connection between the lumen of aorta and gastrointestinal (GI) tract. Secondary AEFs are uncommon but serious complications of both open and endovascular abdominal aortic aneurysm (AAA) repair surgery. Secondary AEF is a result of either graft infection or repeated mechanical stress or a combination of both [1]. Most recent estimates suggest an incidence of 0.8% secondary AEF post endovascular repair (EVAR) [2] and 1% post open repair [3].

Traditionally secondary AEFs have been managed with open surgery: graft explantation, aortic stump closure and axillo-femoral bypasses. Alternative open techniques such as primary closure, *in-situ* bypass grafting using homografts, vein grafts and antimicrobial impregnated grafts are further treatment options with associated varying degrees of success [4]. There is an increasing trend for the use of endografts to occlude fistulas in the emergency setting.

Literature on the subject of management of secondary AEF is limited primarily to case reports and case series. Recent European Society for Vascular Surgery 2020 guidelines on the management of vascular graft and endograft infections advise that for secondary AEF initial treatment with an endograft may be considered, but it is only recommended as a temporary measure [5]. This case series demonstrates a single centre’s experience of successful endovascular techniques that can be used in the emergency management of secondary AEFs.

## CASE SERIES

The first case is a 75-year-old man whose primary procedure was an emergency open AAA repair. Patient had a prolonged postoperative stay on ITU complicated by embolic acute limb ischaemia with resultant above-knee amputation, and upper GI bleed diagnosed endoscopically as secondary to duodenal stress ulcer. Patient spent 2.5 months in-hospital and following rehabilitation returned home.

**Figure 1:**
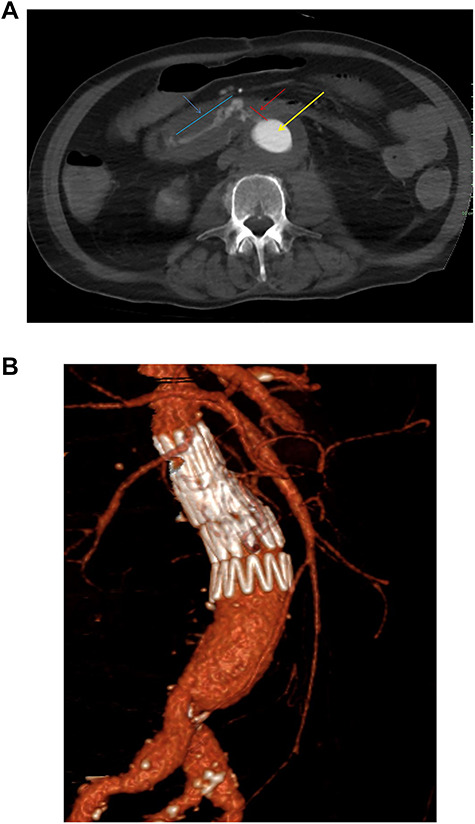
(**a**) Axial thick slice of a CT with IV contrast in the arterial phase shows the lumen of the surgical graft (yellow arrow), clear contrast opacification within the fistula (red line and arrow) and the duodenum (blue line and arrow) consistent with active haemorrhage; (**b**) sagittal reconstruction of a CT with IV contrast in the arterial phase showing the overlapping aortic stent grafts inserted to exclude the AEF from the circulation.

One year after the initial operation, the patient reattended the emergency department with frank haematemesis, rectal bleeding and sepsis in acute haemorrhagic shock. Following resuscitation, an oesophago-gastro duodenoscopy (OGD) revealed fresh blood but no upper GI source of bleeding. Computed tomography (CT) imaging identified a duodenal AEF (Fig. 1a) consistent with communication with the suture line of native aortic-prosthesis. AEF is located 45 mm distal to the lowest (left) branch of the renal artery. The patient successfully underwent emergency relining of the infrarenal aorta into the original graft with a 28 × 43mm COOK® main body extension graft with good overlap (Fig. 1b).

Postoperative imaging revealed complete resolution of AEF and evidence of a small fluid attenuation collection in close relation to the aortic wall with associated 4 cm psoas collection likely secondary to infection. The patient was discharged post-op day 12 on antibiotics with no further complications. Choice of antibiotic therapy determined after discussion with in-hospital microbiology team. Follow up imaging at 3 months postoperatively revealed resolution of the fluid collection, no recurrence of AEF and no aneurysmal sac expansion. Twelve months postprocedure the patient remains well, on lifelong oral antibiotics. This patient will continue on lifelong follow up with 12 monthly cross-sectional imaging. The decision has been made not to proceed to graft explantation due to underlying comorbidities and poor fitness for open repair, alongside patient choice.

**Figure 2:**
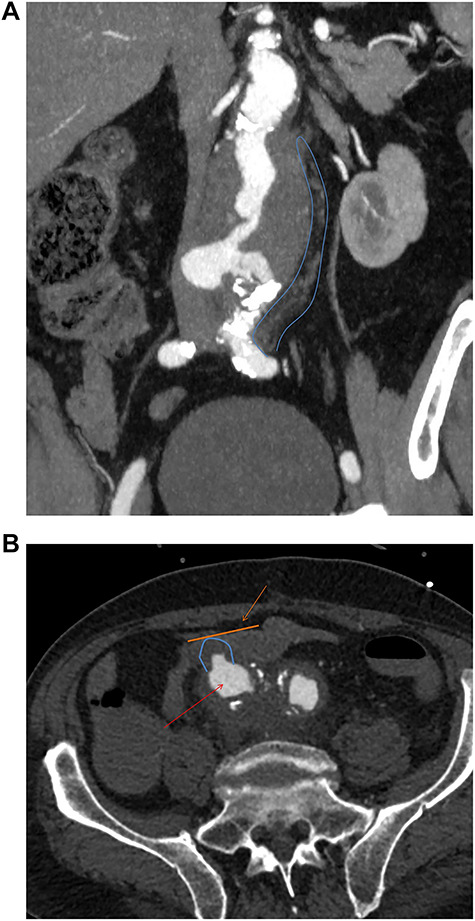
(**a**) Preoperative coronal thick slice of a CT with IV contrast in the arterial phase shows aneurysmal aorta with normal calibre common iliac arteries; however, inflammatory change around the aortic aneurysm is seen extending into the proximal iliac bifurcation (blue outline); (**b**) axial slice of a CT with IV contrast in the arterial phase shows aneurysmal common iliac arteries (red arrow), a clear saccular bulge (blue outline) into an adhered loop of small bowel (orange outline and arrow); the site of the fistulation.

The second case is a 75-year-old gentleman with a background of ulcerative colitis who underwent an elective open tube graft repair for an 8 cm inflammatory aneurysm. In retrospect, inflammatory features are seen to extend distally to the iliac bifurcation on initial imaging (Fig. 2a). Thus, the patient may have benefited from the management of this at the initial surgery.

Fourteen months later, he presented to the emergency department with melaena and haemoglobin of 62 g/l. Following resuscitation an OGD was normal and CT angiography was unable to determine any definitive active bleed but suggested a possible AEF given the close proximity of the third part of duodenum to the aortic graft. Twenty-four hours later, the patient collapsed with a large amount of melaena and frank rectal bleeding in hemorrhagic shock. A new CT scan showed an AEF between distal right common iliac artery and ileum (Fig. 2b) not associated with the suture line of the previous open repair. Of note, this was found to be present on initial emergency imaging when reviewed by a specialist vascular radiologist.

The previous open aneurysm repair was relined with 84 × 24mm COOK® bifurcated endovascular aortic aneurysm repair (EVAR) and 13 mm COOK® limb extension into the right external iliac artery with embolisation of the internal iliac artery. Procedure completed with 24mm COOK® stent extension into contralateral common iliac artery. The patient recovered well from the initial procedure and returned to theatre the next day for laparotomy and resection of the distal ileum adhered to the inflammatory iliac aneurysm. Joint case with the emergency general surgery team at the local hospital performed with extensive washout and small bowel side to side stapled primary anastomosis.

Aside from a prolonged ileus, the patient made a good recovery with intravenous antibiotics and steroids. The patient was discharged on lifelong antibiotics according to local microbiology guidance. At 3 months and 12 months follow up, no evidence of recurrence of AEF, sepsis and no associated mortality. This patient will continue on lifelong surveillance with cross-sectional imaging. Informed consent was obtained from each patient and next of kin when appropriate for this case review.

## DISCUSSION

This case series has provided insight into two different types of secondary AEF and associated endovascular surgery. Bleeding and hemorrhagic shock are common to both presentations of secondary AEF. Diagnostic uncertainty was apparent with differential diagnoses of upper GI bleeding explored initially in both cases. Herald bleeding, in the second case, is defined as an early self-limiting bleed that precedes more devastating continuous haemorrhage and is evidenced in 25–75% of cases of secondary AEF [6]. Arguably, GI bleeding in a patient with previous open or endovascular AAA repair should be considered secondary AEF until proven otherwise. This series suggests that associated sepsis on presentation should heighten suspicion of AEF.

Pathophysiology of AEF differed between cases. The relationship between graft infection and AEF is well documented in the literature [7,8]. Evidence suggests that antibiotic therapy alone is no guarantee of long-term safety in graft infection [3]. Of note, the second case of inflammatory-type iliac aneurysm represents a rare but significant risk factor for secondary AEF [9], particularly when combined with graft infection.

The two cases managed via endovascular approach showed the versatility of EVAR in the successful management of both aortic- and iliac-enteric fistulas. On-site storage of a broad range of aortic stents facilitated flexibility for these emergency cases. Expedient management via endovascular means can be successful in arresting the bleeding and sealing the leak. This is supported by a review article that demonstrated a 30-day in-hospital mortality of 7.1% compared with 33.9% for endovascular vs open surgery, a statistically significant difference (*P* < 0.001) [4]. Furthermore, a review of emergency imaging by a specialist vascular radiologist is imperative in all cases of suspected AEF. This is true in both elective and emergency settings in order to facilitate the best outcomes highlighted by this case series.

Long-term outcomes of both patients post-EVAR repair of secondary AEF have been positive. Neither case has presented to the hospital with recurrent sepsis or recurrent AEF and no associated mortality. Late sepsis is a recognized risk associated with endovascular repair alone due to the likelihood of ongoing underlying graft infection [5]. Evidence suggests that this risk is higher in patients who present with preoperative sepsis [10]. Input from the local microbiology team is paramount to an effective long-term antimicrobial strategy. It remains to be seen whether these cases will develop complications in the future and lifelong follow up with cross-sectional imaging is essential.

These cases differed during their early management with the second case opting for an adjunctive open procedure with resection of the affected portion of the small bowel. Neither case underwent subsequent *in-situ* or extra-anatomical reconstruction. Literature suggests that patients who do not undergo bowel repair have statistically significant increased rates of recurrent sepsis and AEF. One study suggested an incidence of 100% at 12 months [2]. In the first scenario, patient fitness for surgery ruled out adjunctive procedure. This case series supports an individualized approach to AEF management.

This series has highlighted the complexity and diversity of caseload in relation to secondary AEF. Patient anatomy and comorbidities may dictate treatment options. These cases contribute to the increasing body of evidence that endovascular technique can be a safe and effective modality for the management of secondary AEF. COOK® endografts were utilized for the management of secondary AEFs in this case series due to local surgeon preference.

## CONCLUSION

EVAR can be successful in the management of secondary AEF. Careful patient selection, accurate image interpretation, on-site storage of a broad range of aortic stents and expedient management are key factors to successful short- and long-term outcomes.
